# Buffalo-Origin Seneca Valley Virus in China: First Report, Isolation, Genome Characterization, and Evolution Analysis

**DOI:** 10.3389/fvets.2021.730701

**Published:** 2021-10-25

**Authors:** Xia Zhou, Wei-Fang Liang, Guang-Bin Si, Jin-Hui Li, Zhi-Fei Chen, Wei-You Cai, Dian-Hong Lv, Xiao-Hui Wen, Qi Zhai, Shao-Lun Zhai, Ming Liao, Dong-Sheng He

**Affiliations:** ^1^College of Veterinary Medicine, South China Agricultural University, Guangzhou, China; ^2^Institute of Animal Health, Guangdong Academy of Agricultural Sciences, Scientific Observation and Experiment Station of Veterinary Drugs and Diagnostic Techniques of Guangdong Province, Ministry of Agriculture of Rural Affairs, Guangzhou, China; ^3^Key Laboratory of Animal Disease Prevention of Guangdong Province, Guangzhou, China

**Keywords:** Seneca Valley virus, porcine, buffalo, first report, cell lines, host diversity

## Abstract

Pigs are the main host of Seneca Valley virus (SVV), previously known as Senecavirus A (SVA). Pigs affected by SVV have vesicles in the nose, hooves, and limp and may cause death in some severe cases. Occasionally, SVV has also been detected in mice, houseflies, environmental equipment, and corridors in pig farms. Moreover, it was successfully isolated from mouse tissue samples. In this study, an SVV strain (SVA/GD/China/2018) was isolated from a buffalo with mouth ulcers in the Guangdong province of China using seven mammalian cell lines (including BHK-21, NA, PK-15, ST, Vero, Marc-145, and MDBK). The genome of SVA/GD/China/2018 consists of 7,276 nucleotides. Multiple-sequence alignment showed that SVA/GD/China/2018 shared the highest nucleotide similarity (99.1%) with one wild boar-origin SVV strain (Sichuan HS-01) from the Sichuan province of China. Genetic analysis revealed that SVA/GD/China/2018 clustered with those porcine-origin SVV strains. To the best of our knowledge, this is the first report of SVV infection in buffalo, which might expand the host range of the virus. Surveillance should be expanded, and clinical significance of SVV needs to be further evaluated in cattle.

## Introduction

Seneca Valley virus (SVV), previously named Senecavirus A (SVA), is the only member of the *Senecavirus* genus in the *Picornaviridae* family. A typical SVV genome structure is L-VP4-VP2-VP3-VP1-2A-2B-2C-3A-3B-3C-3D. Both the 5′ and 3′ ends are untranslated regions (UTRs) (1). Pigs infected with SVV primarily present with vesicular rash on the nose and coronary band vesicles (2). In severe cases, limping and death occur due to acute myocarditis, heart degeneration, transient fever, and neurological pathology (3). Although SVV was detected in samples as early as 1988 (4), it did not cause any obvious clinical symptoms in pigs before 2008. Sporadic outbreaks of obviously pathogenic SVV occurred between 2008 and 2014. Since 2015, large-scale outbreaks have appeared in the United States, Canada, Brazil, China, Thailand, and Vietnam (2–5). SVV has been detected in mice, houseflies, environmental equipment, and corridors in pig farms and was successfully isolated from mouse tissue samples (6). However, there are no reports of SVV in buffalo. In this study, one SVV strain was first isolated from a buffalo farm in Guangdong, China. The virus was successfully cultured in BHK-21 and NA cells (mouse-origin), PK-15 and ST cells (pig-origin), Vero and Marc-145 cells (monkey-origin), and MDBK cells (bovine-origin). In addition, the viral genome was sequenced and characterized.

## Materials and Methods

### Sample Information

In October 2018, foot and mouth disease (FMD)-like clinical signs, including fever, hoof decay, and limping, were observed in three buffaloes from a buffalo farm (*n* = 80) in Guangdong, China. Three oral swabs were collected by the farm owner and transported to the Animal Disease Diagnostic Center, Institute of Animal Health, Guangdong Academy of Agricultural Sciences, using an insulated container with an ice pack.

### Detection of Potential Pathogens

The viral DNA/RNAs were extracted from the oral swab fluids with the AxyPrep Body Fluid Viral DNA/RNA Miniprep Kit (Corning Life Sciences Co., Ltd., Wujiang, China) and used for reverse transcription PCR to detect foot-and-mouth disease virus (FMDV) (Shenzhen Aodong Inspection and Testing Technology Co., Ltd., Shenzhen, China), vesicular stomatitis virus (VSV) (Guangzhou Vipotion Biotechnology Co., Ltd., Guangzhou, China), bovine viral diarrhea virus (BVDV), bluetongue virus (BTV), and bovine alpha herpesvirus 1 (BoHV-1) according to the reference protocols (7). SVV was also detected using primers SVV-JCF (5′-ATGGTTGGTTTAGCCTGCACAAG-3′) and SVV-JCR (5′-AAGCACGGATGAGACAGAGTTCCAA-3′). One-step RT-PCR (TaKaRa One Step PrimeScript™ RT-PCR Kit, Otsu, Shiga, Japan) was performed with a final reaction volume of 25 μl, containing 12.5 μl 2 × 1 step buffer (Takara, Inc., Shiga, Japan), 0.5 μl PrimeScript One-Step Enzyme Mix (including reverse transcriptase and DNA polymerase), 3 μl viral RNA, 0.5 μl of each primer (10 μmol/l), and 8 μl RNase-free ddH_2_O. PCR conditions were as follows: 50°C for 30 min, 94°C for 5 min followed by 35 cycles of 94°C for 30 s, 59°C for 30 s, and 72°C for 30 s, then the final extension step was 72°C for 5 min. The PCR products were purified with an agarose gel DNA extraction kit (Takara Biomedical Technology, Beijing, Co., Ltd.). The gene cloning experiments were conducted with TaKaRa pMD19-T Vector Cloning Kit (Otsu, Shiga, Japan) and *E. coli* DH5α competent cells (Otsu, Shiga, Japan). In addition, the positive recombinant plasmids were obtained using AxyPrep Plasmid Miniprep Kit (Corning Life Sciences Co., Ltd., Wujiang, China) and sequenced by the Sanger sequencing method (Sangon Biotech Co., Ltd. Shanghai, China).

### The Isolation and Propagation of SVV

Eight cell lines from four different origins were used for virus isolation, which were stored at College of Veterinary Medicine, South China Agricultural University, and Institute of Animal Health, Guangdong Academy of Agricultural Sciences. We used eight cell types of four different origins to isolate the virus. BHK-21 cells, PK-15 cells, ST cells, Vero cells, Marc-145 cells, MDCK cells, and MDBK cells were cultured and passaged in the following growth medium: Dulbecco's modified Eagle's medium (DMEM) (4.5 g/l D glucose, Gibco™, Grand Island, NY, USA). The NA cells were cultured using 1640 medium (RPMI, Gibco™, USA) supplemented with 10% (v/v) fetal bovine serum (FBS, Gibco™, South American). Before the virus inoculation, cell monolayers were washed three times with phosphate-buffered saline without Mg_2+_ and Ca_2+_ [PBS^(−/−)^] to remove FBS and cell metabolites. The virus was added to the cells and incubated at 37°C with a 5% CO_2_ incubator for 1 h. After virus attachment, the virus inoculum was removed and the PBS^(−/−)^ was also used to wash the cell monolayers three times again. Then, the maintenance medium (NA cells: 1640, RPMI, Gibco™, USA, other seven cells: DMEM, 4.5 g/l D glucose, Gibco™, USA) supplemented with 2% (v/v) FBS was added to cells for propagation and passage. The cytopathic effects (CPE) were monitored daily until >90% of the cells showed CPE. The cells were frozen and thawed for three times between −80°C and room temperature, and the virus was filtered with a 0.22-μm filter to remove cell debris and stored at −80°C until further study. Then, the harvested virus solution was identified by RT-PCR and subcultured. SVV was then purified by the virus plaque assay (8). The appropriate plaques were collected and diluted with an appropriate amount of phosphate-buffered saline (PBS, Gibco™). Plaques identified as positive by RT-PCR were inoculated into PK-15 cells and BHK-21 cells, and then passaged and recorded.

### Genome Amplification of SVV

The SVV genome was amplified using seven pairs of primers (Table 1) from one of two positive oral swabs. The target fragments were amplified by the one-step TaKaRa kit (TaKaRa), and the reaction system was 50 μl, including 1 μl PrimeScript One-Step Enzyme Mix, 25 μl 2 × one-step buffer, 1 μl forward primer (10 μmol/l), 1 μl reverse primer (10 μmol/l), 5 μl RNA template, and 17 μl RNase-free ddH_2_O. Cycling conditions were as follows: 50°C for 30 min, pre-denaturation temperature for 5 min followed by 40 cycles of denaturation temperature for 30 s, annealing temperature for 30 s or 15 s, and extension temperature for 30–100 s, separately. A final extension condition was 10 min at 68 or 72°C (Table 1). The target fragments were purified and recovered by an agarose gel DNA extraction kit (TaKaRa). Then each of the seven amplicons of SVV was cloned into pMD19-T. The ligated vector pMD19-T (TaKaRa) was introduced into *E. coli* competent cell DH5α (TaKaRa) for cloning. The extracted plasmids were sent to Sangon Biotech Co., Ltd. (Guangzhou Branch), for sequencing.

**Table 1:** Primers used for genome amplification of SVV.

**Primer**	**Nucleotide sequence (5′-3′)**	**Denaturation temperature**	**Annealing temperature**	**Extension temperature**	**Extension time**	**Product length (bp)**
SVV 1-F	TTTGAAATGGGGGGCTGGGC	95°C	62°C	72°C	30 s	482 bp
SVV 1-R	GTACTCATGGTGGTAGCAGTCACGTGG					
SVV 2-F	ATCACTGAACTGGAGCTCGA	98°C	57°C	68°C	90 s	1,443 bp
SVV 2-R	AGGAGTTCTGTGTCTCTGAGGA					
SVV 3-F	AGTCTCTTGGCACATACTATCGG	98°C	58°C	68°C	100 s	1,614 bp
SVV 3-R	AAGCACGGATGAGACAGAGTTC					
SVV 4-F	TTAAGGTACTGGAGAAGGACGC	98°C	57°C	68°C	90 s	1,385 bp
SVV 4-R	TGGCATTGATCATAGTGGTGAG					
SVV 5-F	TTGGCTCATGATGCCTTCAT	98°C	56°C	68°C	90 s	1,437 bp
SVV 5-R	GTCCAAACTTGTCTAGATTGTTAGGG					
SVV 6-F	CAACAGACCTTCTGGACTTACAC	98°C	57°C	68°C	90 s	1,505 bp
SVV 6-R	AGAGCAGTCCTGATGATCACA					
SVV 7-F	CTCCTTCGAGGCTCTCATCT	98°C	58°C	68°C	35 s	707 bp
SVV 7-R	TCTGTTCCGACTGAGTTCTCC					

### Sequence Analysis of SVV

We constructed a genetic evolutionary tree based on the SVV polyprotein gene with 1,000 bootstrap replicates using MEGA 6.06 software (neighbor-joining method). Full-length nucleotide and amino acid sequence alignments between SVA/GD/China/2018 and other 35 SVV strains published from China and other countries were performed by MegAlign software (DNAStar Lasergene.v7.1) using Clustal W.

## Results

### SVV Detection, Isolation, and Propagation

**Figure 1:**
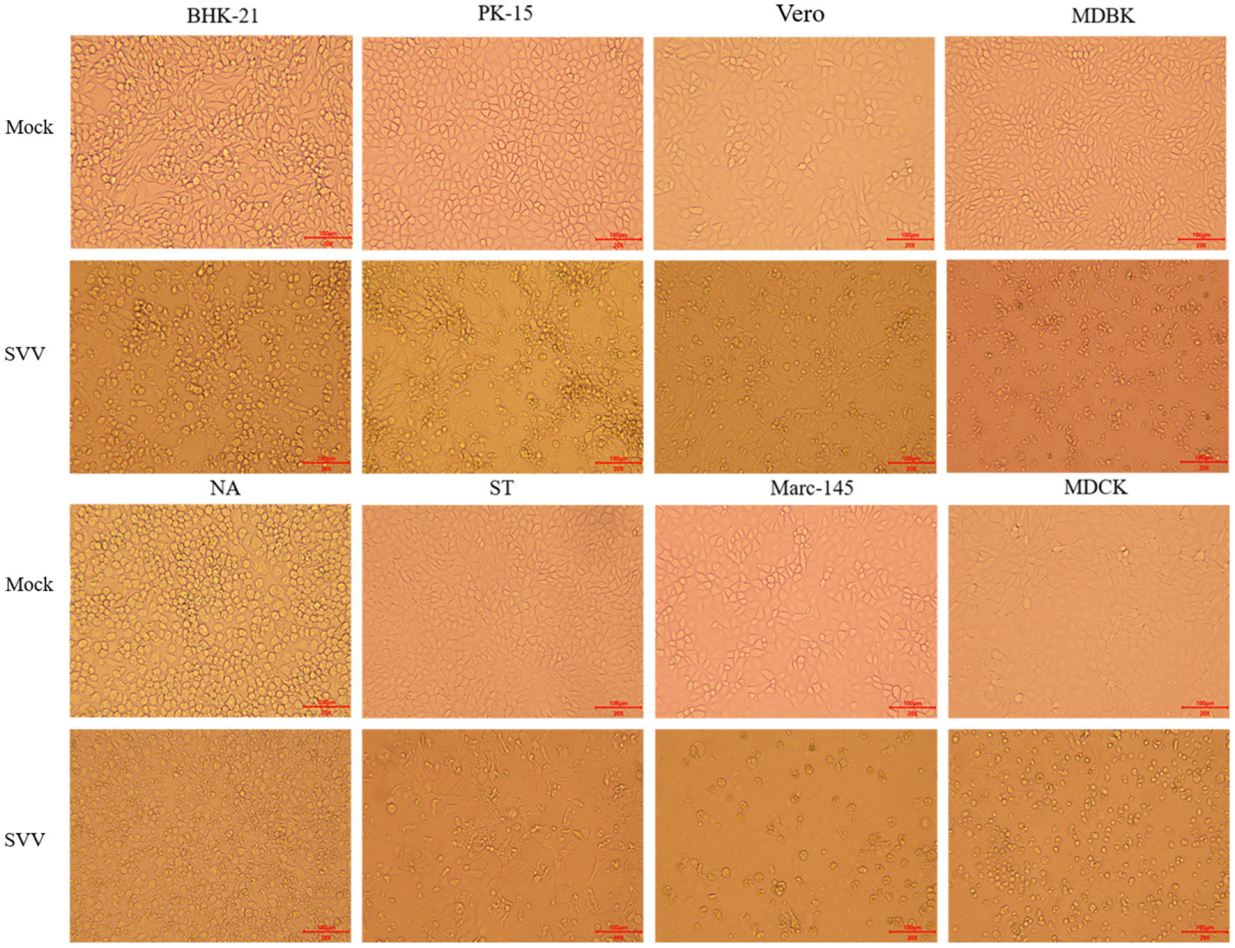
Image of virus isolation with eight different types of cells. The virus was cultured in eight different types of cells, BHK-21 and NA cells, PK-15 and ST cells, Vero and Marc-145 cells, MDBK cells, and MDCK cells. The obvious cytopathic effect, CPE (20×), produced cellular rounding, refraction, cell death, shedding, and floating with BHK-21, PK-15, ST, and Vero cells after 36 h. However, the similar phenomena were not occurred until 96 h in NA cells, Marc-145 cells, and MDBK cells. The virus cannot be cultured for more than four passages with MDCK cells. “Mock” means the negative control cells, and “SVV” means positive control cells that virus propagated.

SVV was detected in two of three samples using RT-PCR, and one of two positive oral swabs was used for genome amplification and sequencing. The buffalo-origin SVV was named SVA/GD/China/2018 (GenBank Accession No. MN615881). The virus was purified and propagated stably to 30 passages in BHK-21 cells, NA cells, PK-15 cells, ST cells, Vero cells, Marc-145 cells, and MDBK cells, but it was propagated only to four passages in MDCK cells in which the SVV cannot be detected after four passages (Figure 1).

### Characterization and Sequence Analysis of Buffalo-Origin SVV

**Figure 2:**
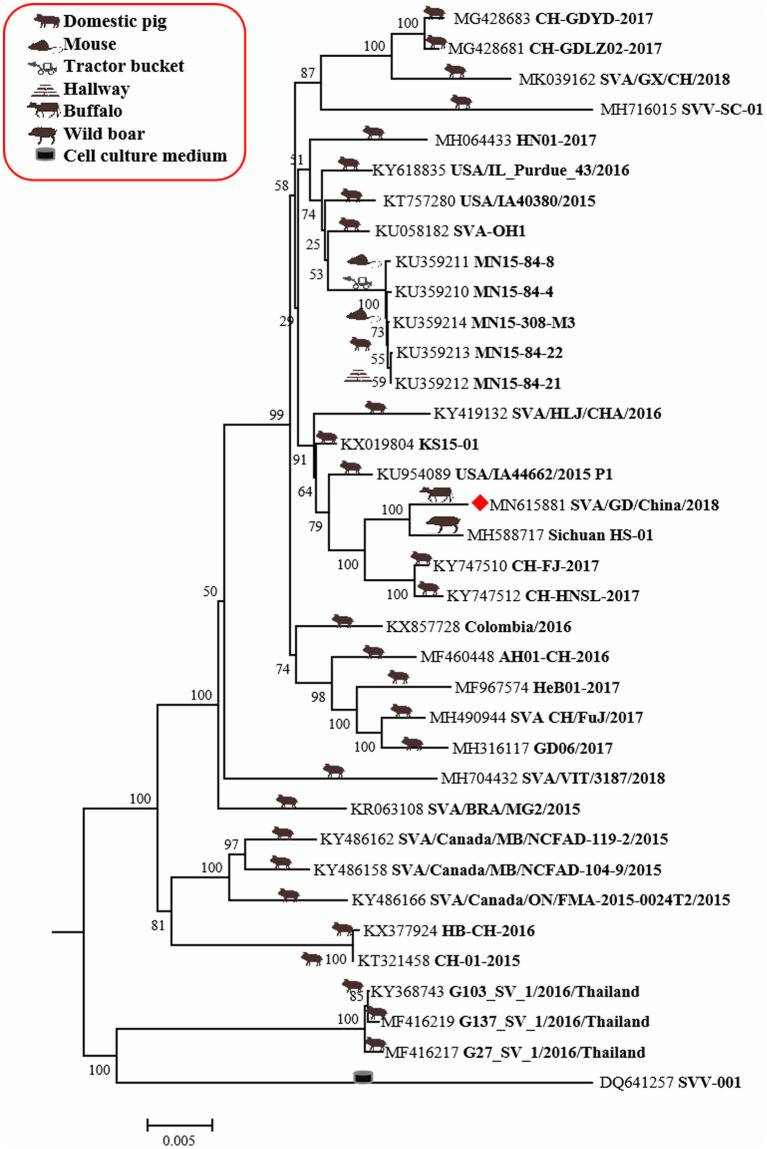
The phylogenetic analysis of SVV based on polyprotein nucleotides. Neighbor-joining tree generated with 1,000 bootstrap samplings (MEGA 6.06). The diamond shape represents the strain in this study. The icon before the accession number indicates the host source of the sequence. At present, only SVA/GD/China/2018 has been derived from diseased buffalo and is in the same branch and closely related to the wild boar-origin strain, Sichuan HS-01.

The genome of SVA/GD/China/2018 consists of 7,276 nucleotides. The sequence analysis showed that the genome similarity of SVA/GD/China/2018 was 93.4–99.1% and the polyprotein similarity was 97.5–99.4% compared with the other 35 known SVV strains (Table 2). Interestingly, SVA/GD/China/2018 shared the highest nucleotide similarity (99.1%) with the wild boar strain (Sichuan HS-01) and the highest polyprotein similarity (99.4%) with the KS15-01 strain. Genetic evolutionary analysis revealed that SVA/GD/China/2018 clustered in the same branch with Sichuan HS-01 from Sichuan, China (Figure 2).

**Table 2:** Partial nucleotide and amino acid percentage identities of the SVA/GD/China/2018 strain compared with other SVV strains.

**Country**	**Area**	**Strain**	**GenBank number**	**Nucleotide similarity**	**Polyprotein similarity**
China	Guangdong	CH-GDYD-2017	MG428683	97.3%	98.8%
	Guangdong	CH-GDLZ02-2017	MG428681	97.4%	98.8%
	Guangdong	GD06/2017	MH316117	97.6%	99.1%
	Guangdong	CH-01-2015	KT321458	96%	98.8%
	Guangxi	SVA/GX/CH/2018	MK039162	96.9%	98.6%
	Heilongjiang	SVA/HLJ/CHA/2016	KY419132	97.9%	99.1%
	Sichuan	Sichuan HS-01	MH588717	99.1%^*^	99.3%
	Sichuan	SVV-SC-01	MH716015	96.4%	98.5%
	Fujian	CH-FJ-2017	KY747510	98.6%	99.1%
	Fujian	SVA CH/FuJ/2017	MH490944	97.6%	99.0%
	Henan	CH-HNSL-2017	KY747512	98.6%	99.0%
	Henan	HN01-2017	MH064433	97.5%	98.8%
	Anhui	AH01-CH-2016	MF460448	97.8%	99.3%
	Hebei	HB01-2017	MF967574	97.6%	98.9%
	Hebei	HB-CH-2016	KX377924	96%	98.7%
USA	Kansas	KS15-01	KX019804	98.5%	99.4%^*^
	Iowa	USA/IA44662/2015 P1	KU954089	98.4%	99.4%^*^

* *Indicates maximum*.

**Figure 3:**
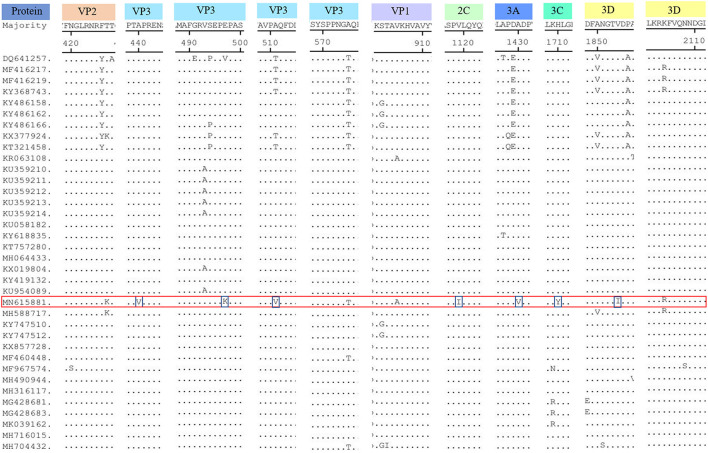
Amino acid sequence alignment of the polyproteins. The SVA/GD/China/2018 strain had seven different amino acids, including 440A (alanine)—V (valine), 497E (glutamic acid)—K (lysine), 511A (alanine)—V (valine) at the VP3 protein; 1119V (valine)—I (isoleucine) at the 2C protein; 1430A (alanine)—V (valine) at the 3A protein; 1710H (histidine)—Y (tyrosine) at the 3C protein, 1854V (valine)—I (Isoleucine) at the 3D protein. For mutations pertaining to amino acids at position 427K (lysine, VP2 protein), 2104R (arginine, 3D protein) was the same as the first wild boar strain, Sichuan HS-01, in China. The “red frame” indicates the buffalo-origin Seneca Valley virus sequence we isolated, and the “blue frame” indicates the different amino acids compared with other sequences.

Compared with the published SVV sequences (Figure 3), SVA/GD/China/2018 was found to have seven unique amino acid substitutions (Figure 2) as follows: 440A (alanine)—V (valine), 497E (glutamic acid)—K (lysine), and 511A (alanine)—V (valine) at the VP3 protein; 1119V (valine)—I (isoleucine) at the 2C protein; 1430A (alanine)—V (valine) at the 3A protein; 1710H (histidine)—Y (tyrosine) at the 3C protein; and 1854V (valine)—I (isoleucine) at the 3D protein.

## Discussion

Viruses such as FMDV (9) and BTV (10), causing symptoms such as ulcers in the mouth and hoof nails, are prevalent in cattle. The results showed that FMDV, BVDV, BTV, VSV, and BoHV-1 were negative, but SVV was detected. This finding was not predicted. Thus, surveillance of SVV in cattle is urgently needed along with animal regression tests.

In order to study the host tropism nature of the virus at the cellular level, eight types of cell lines from four different origins were used to isolate virus from the one of two positive oral swabs. The virus was then successfully purified and cultured to over 30 generations stably in BHK-21 and NA cells of mouse origin, PK-15 and ST cells of pig origin, Vero and Marc-145 cells of monkey origin, and MDBK cells of bovine origin. However, it could be cultured to only four generations in MDCK cells of dog origin, which indicated that MDCK was not the sensitive cell line for SVV. Alternatively, some special measures are needed such as adding trypsin (11). The typical cytopathic effect (CPE), including rounding of cells, refraction, cell death, shedding, and floating, were observed on BHK-21, PK-15, ST, and Vero cells after 36 h, but there was no CPE on another four cells until 96 h (Figure 1). These phenomena indicated that rats, cattle, and monkeys might also be the host of SVV (8), but not dogs. According to previous studies, SVV is pathogenic and causes clinical symptoms in pigs, but it is unclear whether SVV infection could result in visible clinical symptoms in cattle (12). Whether the species supporting replication of SVV in their related cell lines are competent hosts for the virus still needs further confirmation. More studies on related primary cell lines need to be done, such as swine acute diarrhea syndrome coronavirus (SADS-CoV), avian influenza virus, and severe acute respiratory syndrome coronavirus 2 (SARS-CoV-2) (13–15).

The genome of SVA/GD/China/2018 shared lower nucleotide similarity (96–97.6%) and polyprotein similarity (98.8–99.1%) compared with those strains from Guangdong and near Guangdong (Table 2). Interestingly, it shared the highest nucleotide similarity with a wild boar-origin strain (Sichuan HS-01) and the highest polyprotein similarity with the KS15-01 strain, which indicated that SVA/GD/China/2018 was a mutant strain (16). To date, the majority of studies on the genetic relationship analysis of SVV were based on its nucleotide sequence (17–19), while some studies were based on amino acid sequence (20) or on the nucleotide sequence and amino acid sequence simultaneously (21). Because this is the first time to report SVV in buffalo, it is particularly important to investigate and analyze the origin and variation of the virus strain in detail. Therefore, we used both nucleotide sequence and amino acid sequence data of SVV for genetic evolution analysis. Our results showed that the constructed genetic evolutionary tree based on the nucleotide sequence and amino acid sequence of the virus showed a completely consistent trend: SVA/GD/China/2018 is located in the same branch as Sichuan HS-01 from Sichuan, China, indicating that the potential buffalo-derived strain originated from swine. However, the distance between Guangdong and Sichuan is more than 2,000 km long; therefore, further analysis and investigation about how it was transmitted are required.

Interestingly, excluding the possibility of the primers used for amplification to introduce changes in the amplicons, SVA/GD/China/2018 shared the same different amino acids at both 427K (lysine, VP2 protein) and 2104R (arginine, 3D protein) positions, as the first wild boar strain, Sichuan HS-01 (GenBank Accession No. MH588717), in China. In contrast to other porcine strains, they have only one or two mutations. This finding reveals that the virus may have undergone adaptive changes in different hosts (20); however, further investigation is required to elucidate the role of these two residues. VP1 contains a hypervariable region with at least two antigenic sites located at both amino acid 140–160 and amino acid 200–213 sites (22). It has been reported that 228K in VP1, 141–143LDV, and 143–148DGK in VP2 are the primary antigenic sites of FMDV (23). None of these three motifs and antigenic sites of the SVA/GD/China/2018 strain have changed, indicating similar antigenicity and biological characteristics of this strain compared to others (24). The majority of the characteristics are unique, because the different amino acids were located in the VP3 protein. However, further study is required to investigate the effect of these changes.

In summary, we isolated a buffalo-origin SVV strain for the first time and cultured the virus in seven cell lines of different animal origins, and genetic evolution studies revealed the possibility of cross-species transmission of SVV (25).

## Data Availability Statement

The datasets presented in this study can be found in online repositories. The names of the repository/repositories and accession number(s) can be found below: https://www.ncbi.nlm.nih.gov/genbank/, MN615881.

## Ethics Statement

Ethical review and approval were not required for the study because the samples used in the present study were taken from field animals by veterinarians. The samples were taken and submitted to Institute of Animal Health, Guangdong Academy of Agricultural Sciences.

## Author Contributions

XZ and W-FL carried out conceptual and experimental work and wrote the first draft of the manuscript. G-BS, J-HL, Z-FC, and W-YC contributed to the writing and review of the manuscript. D-HL, X-HW, QZ, S-LZ, ML, and D-SH supervised. All authors have approved this manuscript for publication.

## Funding

This study was mostly supported by a grant (Grant No. 2019B020217002) from the Guangdong Provincial Department of Science and Technology, and two grants (Disciplinary Team Construction Program, Grant No. 202122TD and Jinying's Star Talent Program, Grant No. R2018PY-JX003) from Guangdong Academy of Agricultural Sciences, a grant (Grant No. 201906040005) from Guangzhou Science and Technology Bureau, and two grants (Grant Nos. 2021KJ114 and 2021KJ119) from Guangdong Provincial Department of Agriculture and Rural Affairs. Moreover, this study was also partly supported by the Maoming Branch and Zhaoqing Branch, Guangdong Laboratory for Lingnan Modern Agriculture, and Scientific Observation and Experiment Station of Veterinary Drugs and Diagnostic Techniques of Guangdong Province, Ministry of Agriculture of Rural Affairs, and Key Laboratory of Animal Disease Prevention of Guangdong Province.

## Conflict of Interest

The authors declare that the research was conducted in the absence of any commercial or financial relationships that could be construed as a potential conflict of interest.

## Publisher's Note

All claims expressed in this article are solely those of the authors and do not necessarily represent those of their affiliated organizations, or those of the publisher, the editors and the reviewers. Any product that may be evaluated in this article, or claim that may be made by its manufacturer, is not guaranteed or endorsed by the publisher.
